# Analysis of Thymus Extract Action

**Published:** 1915-10

**Authors:** 


**Analysis of Thymus Extract Action**—Rudolf Fischl (Monats. f. Kinderhk., 1914, Bd. XII., Nr. 8) has made a careful investigation of the action of thymus extract in dogs. He finds that the action of thymus extract is very variable. Its effect is shown by a coagulation of blood in the vessels of the nervous system. The same inconstancy is seen in other organic extracts. Vasomotor action is important in its effects on the nervous system. There are individual differences in reaction to the ex tract. No localization of the substance in the spinal cord is observed. The active substance is not in the thymus cells, the cell-free extract being often more active than emulsion of thymus elements. No embolic process is caused by coagulation. In vitro the extract acts as a preventive to coagulation. Specific extracts of other glands such as the suprarenals cause no effect on other non-specific organs. Section of the vagus and the use of atropin has no influence on the action of the extract. Filtration through a Berkfeld filter stops the coagulation effect of the extract. Slow increase of dose causes tolerance to occur, and later much larger amounts can be taken without any bad effects. Anaphylaxis occurs after suspending the use of the extract. The coagulation intensity is variable, and low grades have no bad effect. Previous use of large doses of hirudin destroys the coagulation effect. Pulse is retarded, often irregular; seldom it is not at all influenced. Respiration becomes slow and deeper; occasionally it is irregular; sometimes it is not at all influenced. Terminal convulsions are probably due to anemia. Thrombosis of the vessels of the medulla as a cause of heart and respiratory changes is unlikely. Autopsy shows ecchymoses of the surface of the lungs in rare cases; they are small and result from the terminal convulsions. Changes in the suprarenals are not found.—The American Journal of Obstetrics.

